# The household economic burden for acute coronary syndrome survivors in Australia

**DOI:** 10.1186/s12913-016-1887-3

**Published:** 2016-11-08

**Authors:** Karice K. Hyun, Beverley M. Essue, Mark Woodward, Stephen Jan, David Brieger, Derek Chew, Kellie Nallaiah, Tegwen Howell, Tom Briffa, Isuru Ranasinghe, Carolyn Astley, Julie Redfern

**Affiliations:** 1 The George Institute for Global Health, Sydney Medical School, University of Sydney, Sydney, Australia; 2 Menzies Centre for Health Policy, University of Sydney, Sydney, Australia; 3 Nuffield Department of Population Health, The George Institute for Global Health, University of Oxford, Oxford, UK; 4 Department of Cardiology, Concord Hospital, University of Sydney, Sydney, Australia; 5 Department of Cardiology, Flinders University, Adelaide, Australia; 6 Queensland Health, Brisbane, Australia; 7 School of Population Health, University of Western Australia, Perth, Australia; 8 School of Medicine, University of Adelaide, Adelaide, Australia; 9 South Australia Division, The Heart foundation, Adelaide, Australia; 10 Level 10, King George V Building, 83-117 Missenden Rd, Camperdown, NSW 2050 Australia

**Keywords:** Acute coronary syndrome, Household economic hardship, Out-of-pocket expenditure, Financial burden

## Abstract

**Background:**

Studies of chronic diseases are associated with a financial burden on households. We aimed to determine if survivors of acute coronary syndrome (ACS) experience household economic burden and to quantify any potential burden by examining level of economic hardship and factors associated with hardship.

**Methods:**

Australian patients admitted to hospital with ACS during 2-week period in May 2012, enrolled in SNAPSHOT ACS audit and who were alive at 18 months after index admission were followed-up via telephone/paper survey. Regression models were used to explore factors related to out-of-pocket expenses and economic hardship.

**Results:**

Of 1833 eligible patients at baseline, 180 died within 18 months, and 702 patients completed the survey. Mean out-of-pocket expenditure (*n* = 614) in Australian dollars was A$258.06 (median: A$126.50) per month. The average spending for medical services was A$120.18 (SD: A$310.35) and medications was A$66.25 (SD: A$80.78). In total, 350 (51 %) of patients reported experiencing economic hardship, 78 (12 %) were unable to pay for medical services and 81 (12 %) could not pay for medication. Younger age (18–59 vs ≥80 years (OR): 1.89), no private health insurance (OR: 2.04), pensioner concession card (OR: 1.80), residing in more disadvantaged area (group 1 vs 5 (OR): 1.77), history of CVD (OR: 1.47) and higher out-of-pocket expenses (group 4 vs 1 (OR): 4.57) were more likely to experience hardship.

**Conclusion:**

Subgroups of ACS patients are experiencing considerable economic burden in Australia. These findings provide important considerations for future policy development in terms of the cost of recommended management for patients.

## Background

Acute coronary syndrome (ACS) which includes acute myocardial infarction (MI) and unstable angina (UA) has a high incidence globally [1]. More than 2.5 million hospitalizations are due to ACS worldwide [2]. In Australia, the number of ACS hospitalizations has increased 79 % from 1993 to 2008 for acute MI and 33 % for UA, resulting in 95,000 hospitalizations in 2008 [3]. The direct health care system costs associated with ACS were estimated to be A$1.8 billion and total economic cost of A$17.9 billion in 2009 [4]. As the population ages, grows and the projections for ACS hospitalizations rising, the economic burden, not only to health systems but also to individuals and their households, will inevitably become more pronounced. To better control the potentially substantial economic burden, it is important to understand the current economic wellbeing of the patients. However, this has not been readily explored, especially in high income countries.

Recommended treatment for patients with ACS includes coronary revascularization, adherence to long-term therapies such as: antiplatelet agent(s), beta-blocker, angiotensin-converting enzyme inhibitor, statin and other therapies as appropriate, cardiac rehabilitation services and follow-up appointments with treating physicians [1]. Australian patients benefit from the national health insurance system, Medicare. It can provide ‘bulk billing’, a payment option where a doctor accepts the Medicare benefit as full payment and does not charge patients an extra fee, co-payment system for medical services and listed medications, and further benefits for those with high out-of-pocket health costs (Medicare Safety Net) [5–8]. Despite this, only 72 % of patients continue prescribed medications and 34 % complete cardiac rehabilitation program after hospital discharge [9, 10]. The rate of bulk billing for a specialist appointment is also as low as 29 % [11]. Optimal ACS management involves long-term medical therapies and attendance at programs and appointments to prevent secondary events. Even with government schemes to assist, treatment of ACS may impose an economic burden on patients and contribute to a decrease in the use of medications and health services in general.

Recent research in other chronic diseases in Australia including stroke, chronic obstructive pulmonary disease (COPD) and chronic kidney disease (CKD) indicates that patients experience a significant household economic impact related to their disease condition which has negative consequences for patient outcomes in terms of longer-term treatment adherence and quality of life [12, 13]. There are various reasons for non-adherence to treatment, such as poor understanding of their disease state, ineffective communication between the clinician and the patient, and financial reasons [14]. For ACS survivors, the impact of living with heart disease on the economic well-being of the household remains unclear. Therefore, the aim of this research is to describe the out-of-pocket expenditure associated with ACS and investigate the consequent household economic burden.

## Methods

### Patient cohort

This study was a subgroup analysis of the Australian SNAPSHOT ACS audit. Details relating to the methodology for the broader SNAPSHOT ACS study has been published previously [15]. In brief, all hospitals receiving patients with suspected ACS (including public and private, metropolitan and rural) were identified from their medical records and invited to participate. Patients were eligible to participate in the study if they were admitted overnight with a suspected/confirmed ACS event between May 14th and 27th, 2012. Patients who survived to 18 months after their index admission were followed-up. For the Australian cohort, mortality data at 18 months after index admission was collected via data linkage using the National Death Index. For this sub-study, the household economic status as a part of the 18-month follow-up was collected from two Australian States, New South Wales (NSW) and Queensland (QLD). Human Research Ethics Committee (HREC) approvals were sought for each hospital, including 156 NSW and 122 QLD hospitals. During the 2-week recruitment period, 91 NSW and 61 QLD hospitals recruited suspected ACS patients. Ethics approval for opt-out consent was obtained from all participating centers.

### Data collection

Patients found to be alive at hospital discharge were followed-up by phone/paper survey at approximately 18 months after their index admission. The questionnaire was adapted from a previous study [12]. Paper surveys were posted to the entire cohort and data was entered into a custom built database. Non-responders were followed up by telephone and data was entered directly into the database during the call. The phone survey was conducted using a formal script that was the same as the paper survey. In brief, the questionnaire included information on demographic, education, employment, out-of-pocket expenditure and household economic situation. The follow-up data were then linked to the subset of baseline data from SNAPSHOT ACS. The baseline data collected included demographics, presenting clinical characteristics, receipt of evidence based medication and coronary revascularization. The patient risk at presentation was estimated using the Global Registry of Acute Coronary Events (GRACE) risk score, a score proved to have high capacity to predict mortality [16]. The GRACE risk score was grouped into three risk categories, consistent with the European Society of Cardiology Guideline (low: ≤108, intermediate: 109–140, high >140) [17]. To determine the patient’s socioeconomic status (SES), the postcode of each patient was linked to the Index of Relative Socio-economic Disadvantage (IRSD) score from the Australian Socio-Economic Indexes for Areas (SEIFA) [18]. A low score indicates that the area of residence has relatively greater socioeconomic disadvantage. The IRSD tenths provided by the Australian Bureau of Statistics were used to define five groups for the current analyses.

### Outcomes

The outcomes explored were out-of-pocket expenditure on healthcare and household economic hardship (hardship here after). The out-of-pocket expenditure was the amount spent on managing the medical condition including medical services, medications, ambulance/transport, exercise/allied health, home and self-care assistance and special foods, in the previous 3 months from the time of 18-month follow-up which was not covered by Medicare or private health insurance. A period of 3 months was used to observe the out-of-pocket expenditure as it was considered to be a long enough time period to capture the fluctuation of the expenditure for medical care and treatment over time, but at the same time, a short enough time for patients to recall the expenditures without too much burden. This approach of 3-month recall has been employed in other studies [12, 13]. The expenditure was then converted to monthly expenditure. The collection and analyses of out-of-pocket expenditure were done in Australian Dollars. Hardship was measured using a series of questions about the household’s inability to pay living or medical expense or the use of financial coping strategies (e.g. drew on accumulated savings or sought financial assistance) in order to pay a living expense in the previous 12 months, as defined in a previous study [12]. Variables related to income or socioeconomic status was not used to define hardship as household economic hardship can potentially affect individuals of all socioeconomic status. Patients were categorized into the hardship group if they reported to have difficulties paying at least one expense or used any one or more of the financial coping strategies.

### Statistical analysis

Unadjusted analyses were performed between the patient specific characteristics and the two outcomes: the amount of expenditure and hardship. To compare categorical variables Chi-squared test was used, to compare means between two groups, independent *t*-test was used, and to compare a continuous variable with a skewed distribution and a categorical variable Wilcoxon rank-sum test (for two groups) and Kruskal-Wallis test (for three groups) were used. The zero-inflated negative binomial model was used to derive the relative rates (RRs), the corresponding 95 % confidence intervals (CIs) and the *p*-values for the factors associated with the amount of out-of-pocket expenditure. A multiple-adjusted logistic regression model was used to predict factors associated with hardship. The odds ratios (ORs), the corresponding 95 % CIs were estimated and the *p*-values derived. Candidate variables for both models comprised of those which were statistically significant at α = 0.2 on univariable comparisons. Variables included in the negative binomial regression model predicting the out-of-pocket expenditure were: age (18–59, 60–69, 70–79 and ≥80 years), revascularization from baseline, and private health insurance status, employment status and pensioner concession card at follow-up. Variables included in the logistic regression model to predict hardship: age (categorized as above), SES (IRSD in five groups), GRACE risk categories, smoking status, hypertension and prior cardiovascular disease (CVD) from baseline, and private insurance, employment status, pensioner concession card and out-of-pocket spending (in four groups) at follow-up. Other demographic and clinical characteristics were also explored, however, were not included in the models as they did not meet the level of significance in the univariate analyses. Data were analyzed using SAS 9.4 (SAS Institute Inc. Cary, North Carolina, United States).

## Results

Of 3381 Australian patients from 251 participating hospitals who participated in SNAPSHOT ACS, 1833 had ACS and were admitted to one of the 152 NSW/QLD hospitals and were therefore included in this present analysis. Excluding 180 (10 %) patients who died in hospital and after discharge, 1653 (90 %) NSW/QLD patients were contacted for follow-up at 18 months. Of those, 702 (42 %) responded and agreed to complete the survey relating to household economic status. Patients who have completed the survey had the mean age of 65 years (SD (standard deviation): 13), 431 (61 %) were male, and 263 (37 %) had private health insurance at baseline. The median GRACE risk score for these patients was 94 (IQI (inter-quartile interval): 74–118), 339 (48 %) patients had prior cardiovascular disease, 156 (53 %) had revascularization and 98 (14 %) had an in-hospital event.

### Out-of-pocket expenditure

In total, 92 % (*n* = 614) of patients reported that they had out-of-pocket expenditure. The mean out-of-pocket expenditure in the 3-month period prior to the 18-month follow-up was A$258.06 (SD: A$405.38) per month (Table 1). Of those with out-of-pocket expenditure, 90 % spent on medication, 51 % on medical services (general practitioner, physician specialist, hospitalization and medical test) and 26 % on ambulance/transport. The mean spending for medical services was A$120.18 (SD: A$310.35), medication was A$66.25 (SD: A$80.78), ambulance/transport was A$30.95 (SD: A$130.53) and exercise/allied health was A$15 (SD: A$55).

**Table 1:** Out-of-pocket expenditure (per month)

Items	Mean A$ (SD) for patients with any OOP expenditure on at least one of the items	Median A$ (IQI) for patients with any OOP expenditure on at least one of the items	n (%) patients with any OOP expenditure on the specific item	Median A$ (IQI) for patients with any OOP expenditure on the specific item
Medical service	120.18 (310.35)	10.33 (0.00, 100.00)	277 (51)	100.00 (46.67, 258.33)
Medications	66.25 (80.78)	44.67 (26.67, 73.00)	487 (90)	50.00 (33.33, 80.00)
Ambulance/Transport costs	30.95 (130.53)	0.00 (0.00, 8.33)	142 (26)	40.00 (18.67, 133.33)
Exercise/allied health	16.68 (66.28)	0.00 (0.00, 0.00)	78 (14)	81.67 (40.00, 133.33)
Home and self-care assistance	14.14 (81.90)	0.00 (0.00, 0.00)	50 (9)	84.00 (40.00, 144.00)
Special foods	5.66 (52.95)	0.00 (0.00, 0.00)	19 (4)	66.67 (33.33, 133.33)
Other	4.20 (58.23)	0.00 (0.00, 0.00)	10 (2)	58.33 (30.00, 120.00)
Total expenditure	258.06 (405.38)	126.50 (50.00, 280.00)	540 (100)	126.50 (50.00, 280.00)

Out-of-pocket expenditures are in Australian Dollars

*SD* standard deviation, *IQI* inter-quartile interval, *OOP* out-of-pocket

### Factors associated with out-of-pocket expenditure

Patient factors associated with the amount of out-of-pocket expenditure were predicted. After adjustment, younger patients (18–59 vs. ≥80 years (RR (95 % CI)): 1.80 (1.16, 2.77); 60–69 vs. ≥80 years: 1.63 (1.18, 2.25); and 70–79 vs. ≥80 years: 1.75 (1.29, 2.38); *p* = 0.005) with private health insurance ((RR (95 % CI)): 1.57 (1.30, 1.91); *p* < .0001) were more likely to have higher expenses.

### Household economic hardship

In total, 350 (51 %) respondents reported that they had experienced hardship. That included, 78 (12 %) reporting they were unable to pay for medical consultations/tests, 81 (12 %) for medications, 109 (17 %) for dental appointments, 77 (12 %) for rent or mortgage, 39 (6 %) for meals and 101 (15 %) for utility bills on time. In addition, 221 (34 %) drew on accumulated savings, 61 (9 %) sought financial assistance from welfare/community organization and 71 (11 %) sought financial assistance from friends/family.

### Factors associated with hardship

The characteristics of patients with hardship at 18 months are shown in Table 2. Younger patients (OR for 18–59 vs. ≥80 years: 1.89; 60–69 vs. ≥80 years: 1.64; 70–79 vs. ≥80 years: 0.69), without private health insurance (OR: 2.04), with a pensioner concession card (OR: 1.80), residing in a more disadvantaged area (OR for group 1 vs. 5: 1.77; group 2 vs. 5: 2.30; group 3 vs. 5: 1.31; group 4 vs. 5: 0.92), had history of CVD (OR: 1.47) and with higher out-of-pocket expenditure (OR for group 2 vs. 1: 1.61; group 3 vs. 1: 2.68; and group 4 vs. 1: 4.57) had greater likelihood of experiencing hardship (Table 3).

**Table 2:** Baseline characteristics and in-hospital management for household economic hardship

	Hardship (*n* = 350, 51 %)	No-hardship (*n* = 331, 49 %)	*P*-value
Age, mean (SD)	63 (14)	67 (12)	0.0001
Age			
18–59 years	138 (39)	93 (28)	0.0006
60–69 years	96 (27)	81 (24)	
70–79 years	71 (20)	108 (33)	
80+ years	45 (13)	49 (15)	
Male	224 (64)	197 (60)	0.2287
No private insurance	224 (67)	162 (49)	<.0001
Work status at 18 months			
Employed	79 (24)	90 (28)	<.0001
Unemployed	61 (19)	18 (6)	
Retired	186 (57)	213 (66)	
Education			
No education or primary school only	49 (15)	47 (15)	0.9233
Secondary school only	152 (46)	152 (48)	
TAFE diploma or certificate	74 (22)	65 (20)	
University degree	55 (17)	56 (18)	
Pensioner concession card	222 (67)	195 (59)	0.0425
IRSD (5 groups)			
1 (most disadvantaged)	72 (21)	48 (15)	0.0028
2	104 (30)	72 (22)	
3	80 (23)	83 (25)	
4	57 (16)	81 (24)	
5 (least disadvantaged)	35 (10)	47 (14)	
Medical history			
GRACE risk score (risk categories)			
Low	237 (68)	203 (61)	0. 0528
Intermediate	91 (26)	94 (28)	
High	20 (6)	34 (10)	
Current smoker	74 (21)	36 (11)	0.0003
Hypertension	225 (64)	197 (60)	0.2001
Hyperlipidemia	202 (58)	186 (56)	0.6887
Diabetes	93 (27)	77 (23)	0.3187
Prior CVD^a^	180 (51)	148 (45)	0.0796
Other Comorbidities^b^	63 (18)	49 (15)	0.2607
Diagnosis			
STEMI	28 (8)	38 (11)	0.4650
NSTEMI	62 (18)	60 (18)	
UA	91 (26)	84 (25)	
Other	169 (48)	149 (45)	
In-hospital management			
Cardiac catheterization	140 (40)	142 (43)	0.4425
Revascularization^c^	74/140 (53)	78/142 (55)	0.7270
4 or more EBM at discharge	164 (47)	149 (45)	0.6297
In-hospital events			
In hospital events^d^	52 (15)	44 (13)	0.5577

Data are shown as frequency and proportion (%) unless otherwise specified

*SD* standard deviation, *IRSD* index of relative socio-economic disadvantage, *GRACE* global registry of acute coronary events, *IQI* inter-quartile interval, *CVD* cardiovascular disease, *PCI* percutaneous coronary intervention, *CABG* coronary artery bypass graft, *STEMI* ST elevation myocardial infarction, *NSTEMI* non-ST elevation myocardial infarction, *UA* unstable angina, *EBM* evidence based medications

^a^Prior CVD: prior myocardial infarction, prior peripheral vascular disease, prior stroke, prior PCI, prior CABG or prior atrial fibrillation

^b^Other Comorbidities: prior renal failure, prior major bleeding, prior active cancer, prior dementia or prior impaired mobility

^c^Revascularization: PCI or CABG

^d^In hospital events: myocardial infarction, stroke, heart failure, renal failure, major bleeding or cardiac arrest

**Table 3:** Multiple-adjusted odds ratios and 95 % confidence intervals for household economic hardship vs. no-hardship

Hardship vs. no-hardship		Odds ratio (95 % confidence interval)	*P*-value
Age	18–59 years vs. 80+ years	1.89 (0.77, 4.63)	0.0130
	60–69 years vs. 80+ years	1.64 (0.81, 3.34)	
	70–79 years vs. 80+ years	0.69 (0.38, 1.27)	
No private insurance vs. private insurance		2.04 (1.37, 3.03)	0.0005
Employment status	Unemployed vs. employed	2.06 (0.97, 4.39)	0.1087
	Retired vs. employed	1.01 (0.51, 1.99)	
Pensioner Concession card		1.80 (1.03, 3.18)	0.0406
IRSD^a^	Group 1 vs. 5	1.77 (0.91, 3.45)	0.0043
	Group 2 vs. 5	2.30 (1.23, 4.30)	
	Group 3 vs. 5	1.31 (0.70, 2.45)	
	Group 4 vs. 5	0.92 (0.48, 1.75)	
GRACE risk category	Intermediate vs. low	1.24 (0.76, 2.01)	0.0940
	High vs. low	0.55 (0.25, 1.24)	
Current smoker vs. ex-smoker		1.67 (0.98, 2.82)	0.0576
Hypertension vs. not		1.21 (0.83, 1.76)	0.3144
Prior CVD vs. not		1.47 (1.00, 2.14)	0.0477
Out-of-pocket expenditure^b^	Group 2 vs. 1	1.61 (0.98, 2.64)	<.0001
	Group 3 vs. 1	2.68 (1.62, 4.43)	
	Group 4 vs. 1	4.57 (2.71, 7.70)	

*IRSD* index of relative socio-economic disadvantage, *GRACE* global registry of acute coronary events, *CVD* cardiovascular disease

^a^Group 1 is the most disadvantaged and Group 5 is the least disadvantaged

^b^Group 1 had lowest out-of-pocket expenditure and Group 4 had highest expenditure

## Discussion

This is one of the first known studies to explore the household economic burden for Australians living with heart disease. Results indicate that 50 % of ACS survivors experience substantial household economic burden associated with their illness. On average patients with ACS were spending A$258 per month on health care related out-of-pocket costs. In total, half of the responders to the survey reportedly experienced hardship in the last 12 months prior to the follow-up. Patients who were more likely to have higher out-of-pocket expenditure were those who were older and those with private health insurance. Similar to other studies, hardship was significantly associated with both younger and advanced age, no private health insurance, pensioner concession card, residence of a more disadvantaged area, history of CVD and greater out-of-pocket expenses [19, 20].

Our findings show a substantial monthly out-of-pocket expense following an ACS event, which was greater than the average estimated out-of-pocket health spending in Australia in 2010–11 of A$90 per month, per person [21], the UK in 2013 of $196 per month, per person [22], and the US in 2011 of $101 for 65 years or older patients per month, per person [23]. We also found that a relatively large proportion of patients in our cohort were unable to pay for medical consultations/tests (12 %) and prescription or non-prescription medications (12 %). Similar findings have been reported for other chronic disease areas [12, 13, 24]. For patients with COPD, 25 % reported that they were unable to pay for medical or dental consultations and tests and 18 % for medications, and for CKD patients, 14 % could not for medical appointments and 19 % for medications [12, 13]. The findings suggest that in spite of patients reporting high levels of expenditure on ongoing treatment, there are many patients who report instances where they have foregone treatment due to cost. The policy argument strengthening coverage for out-of-pocket costs is therefore justified as these costs reinforce economic disadvantages in patients with this condition and in addition, represent a significant barrier to optimal management of ACS.

National and international guidelines continue to emphasize the importance of evidence-based medications, along with lifestyle advice and participation in cardiac rehabilitation program for secondary prevention [25]. It is well known that an opposing relationship exists between non-adherence to medications and associated health care cost [26]. It was found that missing doctors’ appointments and sub-optimal communication between clinicians and patient can also lead to non-adherence to medication [27]. Whilst Medicare provides extensive subsidies on prescription medicines and non–hospital medical services and free public hospital care there is a growing recognition that the burden of out-of-pocket costs is significant, particularly for individuals with long term and chronic conditions [28]. The main drivers of these costs are likely to be the need for ongoing multiple medicines and follow-up treatment for people with ACS, and high gap payments (the difference provided by government and the full cost which is covered out-of-pocket) particularly for medical (or physician) specialist consultations.

This study has limitations. As an observational design was used, reporting bias may have been introduced. Also, the response rate to the follow-up study was relatively low; however, assessment of the patient characteristics at baseline and follow-up were similar between NSW/QLD participants and those who were lost to follow-up as well as the rest of the Australian patients. Further, compared to the previous studies that collected similarly detailed and personal data, aiming to find the household economic hardship in other chronic areas, 702 would be considered sizable and well powered to detect important predictors of the household economic hardship such as age, concessional status, and health insurance status [12, 13]. Also, the SNAPSHOT ACS study collected data on a representative and diverse cohort over a 2-week period from more than 200 hospitals across Australia using the opt out methods of consent. Therefore, although the sample size seems modest, the results are generalizable. As patients were asked to provide out-of-pocket expenditure over a 3-month period, it is likely that it was underestimated and be subjected to recall bias. The out-of-pocket expenditure may not have been related to ACS management only, but also for other major comorbidities including diabetes, heart failure, chronic renal failure and major bleeding. However, as ample number of ACS patients manage comorbidities, our estimates reflect the expenditures in reality. Also, when predicting the factors associated with household economic hardship, variables such as prior cardiovascular diseases and out-of-pocket expenditure, which takes into account the costs of comorbidities, were included as independent variables to adjust for comorbidities. In addition, data was collected via self-report and findings need to be considered accordingly.

## Conclusion

In this cohort, we examined the out-of-pocket expenses and household economic situation. The results indicate that along with the high risk medical condition, ACS brings excess out-of-pocket expenditure compared to the national average expenditure. Further, a sizable subset of ACS survivors experiences a substantial household economic burden and that half of the cohort experienced at least once incidence of hardship in the previous 12 months. These findings suggest that out-of-pocket costs pose a significant barrier to optimal management of patients with ACS and more broadly, contribute to exacerbating economic disadvantage amongst household with individuals with chronic disease.

## Appendix

### Participating hospitals

**New South Wales**

Hospital; Armidale Rural Referral Hospital; Ballina District Hospital; Balmain Hospital; Balranald District Hospital; Bankstown Lidcombe Hospital; Baradine MPS; Barham Health Service; Barraba MPS; Bateman’s Bay District Hospital; Bathurst Health Service; Batlow Adelong MPS; Bega Hospital; Bellingen River District Hospital; Belmont Hospital; Berrigan Health Service; Bingara MPS; Blayney District Hospital; Boggabri MPS; Bombala MPS; Boorowa Health Service; Bourke District Hospital; Bowral Hospital; Braidwood MPS; Brewarrina MPS; Broken Hill Base Hospital; Bulahdelah Community Hospital; Byron District Hospital; Calvary Health Care Riverina; Calvary Mater Newcastle; Campbelltown Hospital; Canterbury Hospital; Casino & District Memorial Hospital; Cessnock District Hospital; Cobar Health Service; Coffs Harbour Health Campus; Collarenebri Health Service; Concord Repatriation General Hospital; Condobolin District Hospital; Coolah MPS; Coolamon MPS; Cooma Hospital; Coonamble Health Service; Cootamundra Hospital; Corowa Health Service; Cowra District Hospital; Crookwell District Hospital; Culcairn Health Service; Delegate MPS; Deniliquin Hospital; Denman MPS; Dorrigo MPS; Dubbo Base Hospital; Dunedoo MPS; Dungog Community Hospital; Fairfield Hospital; Finley Hospital; Forbes District Hospital; Gilgandra MPS; Glen Innes Health Service; Gloucester District Health Service; Goodooga Hospital; Gosford Hospital; Goulburn Base Hospital; Grafton Base Hospital; Grenfell MPS; Griffith Base Hospital; Gulargambone MPS; Gulgong Health Service; Gunnedah District Health Service; Hay Hospital; Henty MPS; Hillston Hospital; Inverell Hospital; John Hunter Hospital; Junee MPS; Kempsey Health Campus; Kurri Kurri Hospital; Kyogle Hospital; Lake Macquarie Private Hospital; Leeton Health Service; Lismore Base Hospital; Liverpool Hospital; Lockhart Health Facility; Macksville Health Campus; Maclean Hospital; Maitland Hospital; Manilla MPS; Manly Hospital; Manning Rural Referral Hospital (Taree); Menindee Health Service; Merriwa MPS; Milton-Ulladulla Hospital; Moree District Health Service; Moruya District Hospital; Mudgee Health Service; Mullumbimby Hospital; Murrumburah-Harden Health Service; Muswellbrook District Health Service; Narrabri District Health Service; Narrandera Health Service; Narromine Health Service; Newcastle Private Hospital; Nyngan MPS; Tomaree Community Hospital (Nelson Bay); North Shore Private Hospital; Oberon Health Service; Orange Health Service; Pambula District Hospital; Parkes Health Service; Peak Hill Health Service; Port Macquarie Base Hospital; Prince of Wales Hospital; Prince of Wales Private Hospital; Queanbeyan Hospital; Quirindi Community Hospital; Royal North Shore Hospital; Royal Prince Alfred Hospital; Ryde Hospital; Scott Memorial Hospital (Scone); Shellharbour Hospital; Shoalhaven District Memorial Hospital; Singleton District Hospital; St George Hospital; St George Private Hospital; Strathfield Private Hospital; Sutherland Heart Clinic; Tamworth Rural Referral Hospital; Temora Hospital; Tenterfield Community Hospital; Tibooburra District Hospital; Tocumwal Hospital; Tottenham MPS; Trangie MPS; Trundle MPS; Tullamore MPS; Tumbarumba MPS; Tumut Health Service; Urana MPS; Urbenville Health Service; Vegetable Creek Hospital (Emmaville); Wagga Wagga Base Hospital; Walcha MPS; Walgett Health Service; Warialda MPS; Wee Waa Community Hospital; Wellington Health Service; Werris Creek Health Service; Westmead Private Hospital; Wilcannia MPS; Wilson Community Hospital (Murrurundi); Wollongong Hospital; Wyalong Hospital; Wyong Hospital; Yass District Hospital; Young District Hospital.

**Queensland**

Allamanda Private; Alpha Hospital; Atherton Hospital; Augathella Hospital; Ayr Hospital; Babinda Hospital; Bamaga Hospital; Baralaba Hospital; Barcaldine Hospital; Beaudesert Hospital; Biggenden Hospital; Biloela Hospital; Blackall Hospital; Blackwater Hospital; Boonah Hospital; Bowen Hospital; Bundaberg Friendly; Bundaberg Hospital; Caboolture Hospital; Cairns Base Hospital; Cairns Private; Caloundra Hospital; Capricorn Coast Hospital; Charleville Hospital; Charters Towers Hospital; Cherbourg Hospital; Childers Hospital; Chinchilla Hospital; Clermont Hospital; Collinsville Hospital; Cooktown Hospital; Cloncurry Hospital; Dalby Hospital; Dirranbandi Hospital; Doomadgee Hospital; Eidsvold Hospital; Emerald Hospital; Esk Hospital; Dysart Hospital; Gatton Hospital; Gayndah Hospital; Gin Gin Hospital; Gladstone Hospital; Gold Coast Hospital; Goondiwindi Hospital; Greenslopes Private; Gympie Hospital; Hervey Bay Hospital; Hillcrest Private Hospital; Hughenden Hospital; Ingham Hospital; Inglewood Hospital; Innisfail Hospital; Ipswich Hospital; John Flynn; Jandowae Hospital; Kilcoy Hospital; Joyce Palmer Hospital; Julia Creek Hospital; Laidley Hospital; Logan Hospital; Longreach Hospital; Mackay Base Hospital; Maleny Hospital; Mareeba Hospital; Maryborough Hospital; Mater Adult Public Hospital; Mater Private Brisbane; Mater Private Mackay; Mater Private Rockhampton; Mater Private Townsville; Miles Hospital; Millmerran Hospital; Mitchell Hospital; Monto Hospital; Moranbah Hospital; Mornington Island Hospital; Mossman Hospital; Mount Isa Hospital; Mount Morgan Hospital; Moura Hospital; Mundubbera Hospital; Mungindi Hospital; Murgon Hospital; Nambour Hospital; Nambour Selengor; Nanango Hospital; Normanton Hospital; Princess Alexandra Hospital; Proserpine Hospital; Queen Elizabeth II Jubilee Hospital; Quilpie Hospital; Redcliffe Hospital; Redland Hospital; Richmond Hospital; Rockhampton Base Hospital; Roma Hospital; Royal Brisbane & Women’s Hospital; Sarina Hospital; St Andrew’s Private Toowoomba; St Andrew’s War Memorial, Brisbane; St George Hospital; St Vincent’s Hospital; Springsure Hospital; Stanthorpe Hospital; Sunshine Coast Private Hospital; Surat Hospital; Tara Hospital; Texas Hospital; Theodore Hospital; The Prince Charles Hospital; Thursday Island Hospital; Toowoomba Hospital; Townsville Hospital; Tully Hospital; Warwick Hospital; Weipa Hospital; Winton Hospital; Wesley Hospital; Woorabinda Hospital; Wynnum Hospital; Yarrabah Hospital.

## Abbreviations

ACS: Acute coronary syndrome
CI: Confidence interval
CKD: Chronic kidney disease
COPD: Chronic obstructive pulmonary disease
CVD: Cardiovascular disease
GRACE: Global registry of acute coronary events
IQI: Inter-quartile interval
IRSD: Index of relative socio-economic disadvantage
MI: Myocardial infarction
NSW: New South Wales
OR: Odds ratio
QLD: Queensland
RR: Relative rate
SD: Standard deviation
SEIFA: Socio-economic indexes for areas
SES: Socioeconomic status
UA: Unstable angina
